# A Metric on the Space of Partly Reduced Phylogenetic Networks

**DOI:** 10.1155/2016/7534258

**Published:** 2016-06-23

**Authors:** Juan Wang

**Affiliations:** School of Computer Science, Inner Mongolia University, Hohhot 010021, China

## Abstract

Phylogenetic networks are a generalization of phylogenetic trees that allow for the representation of evolutionary events acting at the population level, such as recombination between genes, hybridization between lineages, and horizontal gene transfer. The researchers have designed several measures for computing the dissimilarity between two phylogenetic networks, and each measure has been proven to be a metric on a special kind of phylogenetic networks. However, none of the existing measures is a metric on the space of partly reduced phylogenetic networks. In this paper, we provide a metric, *d*_*e*_-distance, on the space of partly reduced phylogenetic networks, which is polynomial-time computable.

## 1. Introduction

Phylogenies reveal the history of evolutionary events of a group of species, and they are central to comparative analysis methods for testing hypotheses in evolutionary biology [1]. Computing the distance between a pair of phylogenies is very important for understanding the evolutionary history of species.

A metric *d* on a space *S* satisfies four properties for all *a*, *b*, *c* ∈ *S*:

(I) *d*(*a*, *b*) ≥ 0 (nonnegative property);
(II) *d*(*a*, *b*) = 0 if and only if *a* = *b* (separation property);
(III) *d*(*a*, *b*) = *d*(*b*, *a*) (symmetry property);
(IV) *d*(*a*, *b*) + *d*(*b*, *c*) ≥ *d*(*a*, *c*) (triangle inequality).

Phylogenetic network can represent reticulate evolutionary events, such as recombinations between genes, hybridization between lineages, and horizontal gene transfer [2–5]. For the comparison of phylogenetic networks, there are many metrics on the restricted subclasses of networks including the tripartition metric on the space of tree-child phylogenetic networks [6–9], the *μ*-distance on the space of tree-sibling phylogenetic networks [10], and the *m*-distance on the space of reduced phylogenetic networks [11]. Later the *m*-distance was also proved to be a metric on the space of tree-child phylogenetic networks, semibinary tree-sibling time consistent phylogenetic networks, and multilabeled phylogenetic trees [12–15].

For any rooted phylogenetic network *N*, we can obtain its reduced version by removing all nodes in maximal convergent sets (will be discussed in the following) and all the nodes, with indegree 1 and outdegree 1, from *N*. The reduced versions of all rooted phylogenetic networks form the space of reduced phylogenetic networks (*m*-distance, defined by Nakhleh, is on this space). In this paper, we will discuss the partly reduced version of a phylogenetic network by removing the nodes in a part of the convergent sets and all the nodes, with indegree 1 and outdegree 1, from the phylogenetic network. The partly reduced versions of all rooted phylogenetic networks form the space of partly reduced phylogenetic networks. Then we will introduce a novel metric on the space of partly reduced phylogenetic networks. The space is not the space of rooted phylogenetic networks, but it is the largest space on which a polynomial-time computable metric has been defined so for. The papers [16, 17] have proved that the isomorphism for rooted phylogenetic networks is graph isomorphism-complete. Unless the graph isomorphism problem belongs to *P*, there is no hope of defining a polynomial-time computable metric on the space of all rooted phylogenetic networks. However, our paper's aim is mainly to find a larger space on which a polynomial-time computable metric can be defined such that the space is closer to the space of rooted phylogenetic networks.

## 2. Preliminaries

Let *N* = (*V*, *E*) be a directed acyclic graph, or DAG for short. We denote the indegree of a node *u* as indeg(*u*) and the outdegree of *u* as outdeg(*u*). We will say that a node *u* is a* tree node* if indeg(*u*) ≤ 1. Particularly, *u* is a* root* of *N* if indeg(*u*) = 0 of *N*. If a single root exists, we will say that the DAG is* rooted*. We will say that a node *u* is a* reticulate node* if indeg(*u*) ≥ 2. A tree node *u* is a* leaf* if outdeg(*u*) = 0. A node is called an* internal node* if its outdeg ≥ 1. For a DAG *N* = (*V*, *E*), we will say that *v* is a* child* of *u* if (*u*, *v*) ∈ *E*; in this case, we will also say that *u* is a* parent* of *v*. Note that any tree node has a single parent, except for the root of the graph. Whenever there is a directed path from a node *u* to *v*, we will say that *v* is a* descendant* of *u* or *u* is an* ancestor* of *v*.

The* height* of a node is the length of a longest path starting at the node and ending in a leaf. The absence of cycles implies that the nodes of a DAG *N* can be stratified by means of their heights: the nodes of height 0 are the leaves; if a node has height *a* > 0, then all its children have heights that are smaller than *a* and at least one of them has height exactly *a* − 1.

The* depth* of a node is the length of a longest path starting at the root and ending in the node. Similarly, the absence of cycles implies that the nodes of a DAG *N* can also be stratified according to their depths: the node of depth 0 is the root; if a node has depth *b* > 0, then all its parents have depths that are smaller than *b* and at least one of them has depth exactly *b* − 1.

Let *𝒳* be a set of taxa. A rooted phylogenetic network *N* on *𝒳* is a rooted DAG such that

(i) no tree node has outdeg 1;
(ii) its leaves are labeled by *𝒳* by a bijective mapping *f*.

We use the notation *N* = ((*V*, *E*), *f*) (or *N* = (*V*, *E*)) for the rooted phylogenetic network *N* and the notation *V*_*N*_ for its leaf set.

**Definition 1 .**

Two rooted phylogenetic networks *N*_1_ = ((*V*_1_, *E*_1_), *f*_1_) and *N*_2_ = ((*V*_2_, *E*_2_), *f*_2_) are isomorphic if and only if there is a bijection *G* from *V*_1_ to *V*_2_ such that

(i) (*u*, *v*) is an edge in *E*_1_ if and only if (*G*(*u*), *G*(*v*)) is an edge in *E*_2_;
(ii) *f*_1_(*w*) = *f*_2_(*G*(*w*)) for all *w* ∈ *V*_*N*_1__.

Moret et al. (2004) discussed the concept of reduced phylogenetic networks from a reconstruction standpoint. Subsequently, we briefly review the concept of reduced phylogenetic networks and introduce a new definition of partly reduced phylogenetic networks. In the following section, we present a metric on the space of all partly reduced phylogenetic networks. First we review the concept of a maximal convergent set that has been given in [7, 11].

**Definition 2 .**

Given a network *N* = (*V*, *E*), we say that a set *U* of internal nodes in *V* is convergent if |*U* | ≥ 2 and

 every leaf reachable from some node in *U* is reachable from all nodes in *U*.

If there is no convergent set *U*_0_ containing *U* except *U* itself, we say that *U* is a maximal convergent set.

Here the leaf set reachable from the nodes in a convergent set *U* is called the leaf set of *U*.

**Figure 1:**
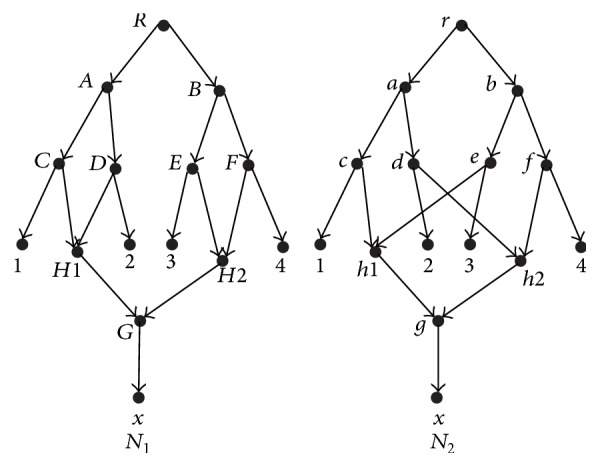
Networks *N*_1_ and *N*_2_ from refinements (1) and (2) in Table 1 in [11]. *H*1 and *H*2 (resp., *h*1 and *h*2) are the reticulate nodes,* A*~*G* (resp.,* a*~*g*); *H*1 and *H*2 (resp., *h*1 and *h*2) as well as the root *R* (resp., *r*) are the internal tree nodes in network *N*_1_ (resp., *N*_2_).

We will take Figure 1 as an example in the following. The two networks *N*_1_,  *N*_2_ on {1, 2, 3, 4, *x*} are adapted from refinements (1) and (2) in Table 1 in [11].

**Example 3 .**

Consider the networks in Figure 1. The set {*H*1, *H*2, *G*} is the only maximal convergent set of *N*_1_ and the set {*h*1, *h*2, *g*} is the only maximal convergent set of *N*_2_.

For a phylogenetic network *N* = ((*V*, *E*), *f*) on *𝒳*, the reduced version of *N* can be obtained by the following reduction procedures:

(1) For each maximal pendant subtree (i.e., the maximal clade that includes no reticulate nodes) *t*, rooted at node *r*_*t*_, create a new node *h*_*t*_ and an edge (*p*_*t*_, *h*_*t*_), where *p*_*t*_ is the parent of *r*_*t*_, delete the edge (*p*_*t*_, *r*_*t*_) and the subtree *t*, and label *h*_*t*_ as *t*. Then we denote the resulting network as *N*_0_.
(2) Repeat the following two steps on *N*_0_ until no change occurs:
  (I) For each maximal convergent set *U* with leaf set *L*_*U*_⊆*V*_*N*_0__, remove all nodes and edges on the paths from a node in *U* to the parent of leaf in *L*_*U*_, including all nodes in *U* and excluding the parent of leaf in *L*_*U*_. For each edge (*p*, *v*), where *p* lies outside the deleted set and *v* lies inside the deleted set, replace it with a set of edges {(*p*, *q*):  *q* is the parent of leaf in *L*_*U*_}.
  (II) For each node *w* in the network, with indeg(*w*) = outdeg(*w*) = 1, remove the edges (*u*, *w*), (*w*, *v*) and the node *w*, add an edge (*u*, *v*), where *u* is the parent of *w* and *v* is the child of *w*. Repeat this step until no such node can be removed.
(3) Replace each leaf labeled by the subtree *t* by its root *r*_*t*_.

**Figure 2:**
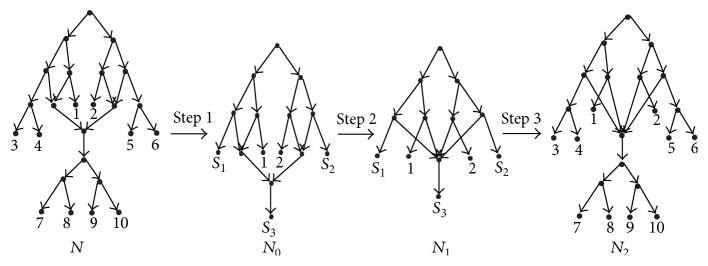
The rooted phylogenetic network *N* is on {1,2, 3,4, 5,6, 7,8, 9,10}. *N*_0_, *N*_1_, and *N*_2_ are the networks obtained by applying each one of the three reduction procedures to *N*, respectively.

**Figure 3:**
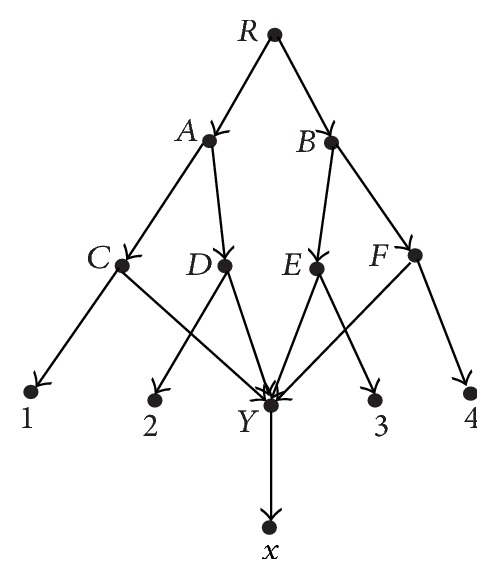
The reduced version of the networks in Figure 1.

Figure 2 shows the results of applying the reduction procedures to the network *N*. For the networks in Figure 1, their reduced versions are the same (see Figure 3). The reduced versions of all rooted phylogenetic networks form the space of reduced phylogenetic networks. Nakhleh has introduced a polynomial-time computable metric on this space [11]. In order to enlarge the space in which a polynomial-time computable metric can be defined, we will introduce a new metric and a new space that contains the space of reduced phylogenetic networks.

**Definition 4 .**

Given a network *N* = (*V*, *E*), let *𝒫*(*v*) be the set of parents of a node *v* in *V*. We say that *U* ⊂ *V* is a super convergent set, if

(i) *U* is a convergent set;
(ii) *𝒫*(*u*_1_) = *𝒫*(*u*_2_) for any two nodes *u*_1_, *u*_2_ ∈ *U*;
(iii) *𝒫*(*u*) is a convergent set for a node *u* ∈ *U*, if |*𝒫*(*u*)| ≥ 2.

**Example 5 .**

**Figure 4:**
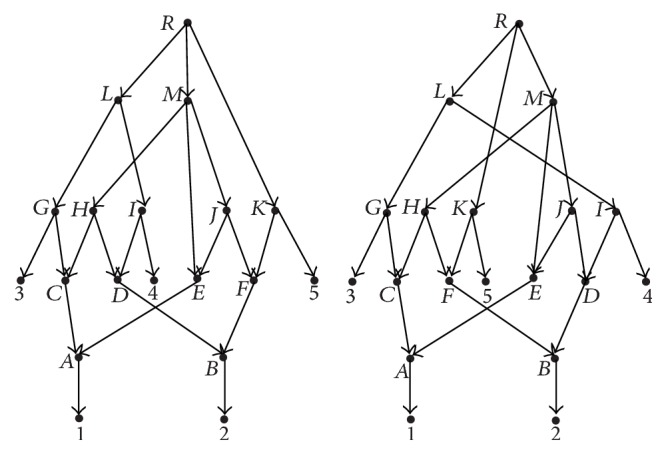
Networks *N*_1_ and *N*_2_ are not isomorphic.

The set {*H*, *J*} is the only superconvergent set for any one network in Figure 4, while the networks in Figure 1 have no superconvergent set.

We will obtain the new reduction procedures, called partial reduction procedures, from the above reduction procedures by just processing superconvergent sets rather than maximal convergent sets in step (I) of step (2). After applying the partial reduction procedures to a rooted phylogenetic network *N*, the partly reduced version of *N* is obtained. The partly reduced versions of all rooted phylogenetic networks form the space of partly reduced phylogenetic networks. This space contains the space of reduced phylogenetic networks, but they are not identical. Next we will introduce a polynomial-time computable metric for the partly reduced phylogenetic networks.

We begin with the notion of node semiequivalence. For the sake of simplicity, we will hereafter refer to the rooted phylogenetic networks as the networks.

## 3. A Metric

**Definition 6 .**

Given a network *N* = ((*V*, *E*), *f*), we say that two nodes *u*, *v* ∈ *V* (not necessarily different) are semiequivalent, denoted by *u*≜*v*, if

(i) *u*, *v* ∈ *V*_*N*_ and *f*(*u*) = *f*(*v*) or
(ii) node *u* has *k*  (≥1) children *u*_1_, *u*_2_,…, *u*_*k*_; node *v* has *k* children *v*_1_, *v*_2_,…, *v*_*k*_, and *u*_*i*_≜*v*_*i*_ for 1 ≤ *i* ≤ *k*.

By the definition, it follows that the semiequivalence of nodes is an equivalence relation; that is, it is reflexive, symmetric, and transitive, and the semiequivalent nodes must have the same height.

**Example 7 .**

Consider the network *N*_1_ in Figure 1. For any node *u* ∈ *V*_1_∖{*H*1, *H*2}, *u* is only semiequivalent to *u* itself, while the nodes *H*1 and *H*2 are semiequivalent.

**Property 1 .**

**Figure 5:**
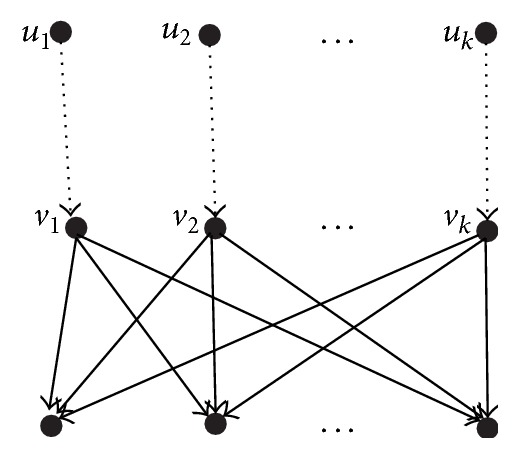
The topology relation of semiequivalent nodes.

If *u*_1_, *u*_2_,…, *u*_*k*_ are semiequivalent from the network *N* = ((*V*, *E*), *f*), then *u*_1_, *u*_2_,…, *u*_*k*_ are the same nodes or there are the nodes *v*_1_ (*u*_1_ or a descendant of *u*_1_), *v*_2_ (*u*_2_ or a descendant of *u*_2_),…, *v*_*k*_ (*u*_*k*_ or a descendant of *u*_*k*_) such that *v*_1_, *v*_2_,…, *v*_*k*_ have the same children. See Figure 5.

**Proof**

We use induction on the height *a* of *u*_1_ to prove it. If *a* = 0, obviously *u*_1_, *u*_2_,…, *u*_*k*_ are the only leaf. Thus, in this case, the property holds. We assume that the result is tenable when *a* ≤ *n*, and let *a* = *n* + 1. Then the children of *u*_1_, *u*_2_,…, *u*_*k*_ are semiequivalent, respectively (let the children of *u*_*i*_ be *a*_*i*1_, *a*_*i*2_,…, *a*_*il*_ for 1 ≤ *i* ≤ *k*; then *a*_1*j*_, *a*_2*j*_,…, *a*_*kj*_ are semiequivalent for 1 ≤ *j* ≤ *l*), and their height is at most *n* by the property of node height. By the induction hypothesis, the children of *u*_1_, *u*_2_,…, *u*_*k*_ satisfy the property. The descendants of children of *u*_1_, *u*_2_,…, *u*_*k*_ are the descendants of *u*_1_, *u*_2_,…, *u*_*k*_. Thus, the property holds.

**Definition 8 .**

Given a network *N* = (*V*, *E*), we say that two nodes *u*, *v* ∈ *V* (not necessarily different) are equivalent, denoted by *u* ≡ *v*, if *u*≜*v*, and

(i) *u*, *v* are the root or
(ii) node *u* has *l*  (≥1) parents *u*_1_, *u*_2_,…, *u*_*l*_; node *v* has *l* parents *v*_1_, *v*_2_,…, *v*_*l*_, and *u*_*i*_ ≡ *v*_*i*_ for 1 ≤ *i* ≤ *l*.

For any node *u* in *N*, it is equivalent to itself. The equivalence of nodes is also an equivalence relation. The equivalent nodes have the same height and depth.

**Example 9 .**

Consider the network *N*_1_ in Figure 1. For any node *u* ∈ *V*_1_, it is equivalent to itself. Consider the network *N*_1_ in Figure 4. For any node *u* ∈ *V*_1_∖{*H*, *J*}, it is equivalent to itself, while the nodes *H* and *J* are equivalent to each other.

**Property 2 .**

**Figure 6:**
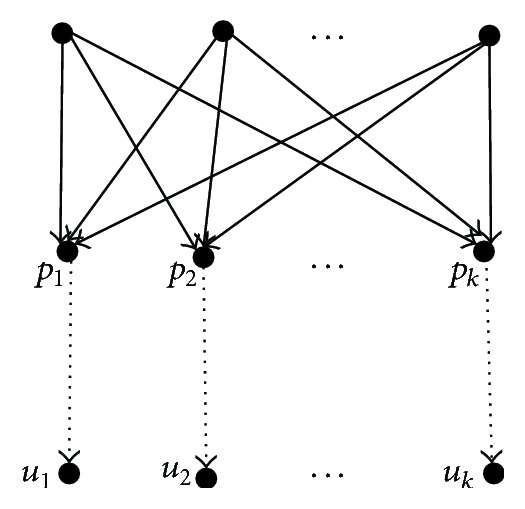
The topology relation of equivalent nodes.

If *u*_1_, *u*_2_,…, *u*_*k*_ are equivalent in the network *N* = ((*V*, *E*), *f*), then *u*_1_, *u*_2_,…, *u*_*k*_ are the same nodes or there are the nodes *p*_1_ (*u*_1_ or an ancestor of *u*_1_), *p*_2_  (*u*_2_ or an ancestor of *u*_2_),…, *p*_*k*_ (*u*_*k*_ or an ancestor of *u*_*k*_) such that *p*_1_, *p*_2_,…, *p*_*k*_ have the same parents. See Figure 6.

**Proof**

We use induction on the depth *b* of *u*_1_ to prove it. If *b* = 0, then *u*_1_, *u*_2_,…, *u*_*k*_ are the unique root node. Thus, in this case, the property holds. We assume that the result is tenable when *b* ≤ *n*, and let *b* = *n* + 1. Then the parents of *u*_1_, *u*_2_,…, *u*_*k*_ are equivalent, respectively (let the parents of *u*_*i*_ be *a*_*i*1_, *a*_*i*2_,…, *a*_*il*_ for 1 ≤ *i* ≤ *k*; then *a*_1*j*_, *a*_2*j*_,…, *a*_*kj*_ are equivalent for 1 ≤ *j* ≤ *l*), and their depth is at most *n* by the property of node depth. By the induction hypothesis, the parents of *u*_1_, *u*_2_,…, *u*_*k*_ satisfy the property. The ancestors of the parents of *u*_1_, *u*_2_,…, *u*_*k*_ are the ancestors of *u*_1_, *u*_2_,…, *u*_*k*_. Thus, the property holds.

In this paper, we are mainly concerned with comparing networks; the notion of node semiequivalence and equivalence will be extended to nodes from two different networks, as established in the semiequivalence and equivalence mapping of Definitions 10 and 13, respectively.

Given a set *V*, we use *P*(*V*) to denote the set of all subsets of *V*.

**Definition 10 .**

Let *N*_1_ = ((*V*_1_, *E*_1_), *f*_1_) and *N*_2_ = ((*V*_2_, *E*_2_), *f*_2_) be two networks on *𝒳*. We define the semiequivalence mapping between *N*_1_ and *N*_2_, *h* : *V*_1_ → *P*(*V*_2_), such that *v* ∈ *h*(*u*), for *u* ∈ *V*_1_ and *v* ∈ *V*_2_, if

(i) *u* ∈ *V*_*N*_1__, *v* ∈ *V*_*N*_2__, and *f*_1_(*u*) = *f*_2_(*v*) or
(ii) node *u* has *k*  (≥1) children *u*_1_, *u*_2_,…, *u*_*k*_; node *v* has *k* children *v*_1_, *v*_2_,…, *v*_*k*_, and *v*_*i*_ ∈ *h*(*u*_*i*_) for 1 ≤ *i* ≤ *k*.

Further, while inequation |*h*(*u*_1_)| ≤ 1 holds in phylogenetic trees, it is not always the case for general phylogenetic networks.

**Example 11 .**

Consider the networks in Figure 1. *h* is a semiequivalence mapping between *N*_1_ and *N*_2_. For the reticulate nodes *H*1 and *H*2 in *N*_1_, *h*(*H*1) = {*h*1, *h*2} and *h*(*H*2) = {*h*1, *h*2}. For the other nodes in *N*_1_, *h*(*A*) = {*a*},  *h*(*B*) = {*b*},…, *h*(*G*) = {*g*},  *h*(1) = {1},…, *h*(4) = {4},  *h*(*x*) = {*x*}, and *h*(*R*) = {*r*}.

**Theorem 12 .**

Let *N*_1_ = ((*V*_1_, *E*_1_), *f*_1_) and *N*_2_ = ((*V*_2_, *E*_2_), *f*_2_) be two networks on *𝒳*, and let *u*_1_, *u*_2_ be two nodes in *V*_1_ and *h* a semiequivalence mapping between *N*_1_ and *N*_2_. Assume that *h*(*u*_1_) ≠ *∅* and *h*(*u*_2_) ≠ *∅*. Then, *u*_1_≜*u*_2_ if and only if *v*_1_≜*v*_2_, for *v*_1_ ∈ *h*(*u*_1_) and *v*_2_ ∈ *h*(*u*_2_).

**Proof**

For the “only if” direction, let *v*_1_ ∈ *h*(*u*_1_), *v*_2_ ∈ *h*(*u*_2_), and *u*_1_≜*u*_2_. Obviously, *u*_1_,  *u*_2_,  *v*_1_, and *v*_2_ have the same height *a*. Then, we use induction on such height *a* to prove *v*_1_≜*v*_2_. In particular, if *a* = 0, that is, *u*_1_, *u*_2_ ∈ *V*_*N*_1__, and *f*_1_(*u*_1_) = *f*_1_(*u*_2_), then *v*_1_, *v*_2_ ∈ *V*_*N*_2__ and *f*_2_(*v*_1_) = *f*_1_(*u*_1_) = *f*_1_(*u*_2_) = *f*_2_(*v*_2_). Thus, in this case, *v*_1_≜*v*_2_. We assume that the result is tenable when *a* ≤ *n*, and let *a* = *n* + 1. We assume that node *u*_1_ has *k* children *p*_1_, *p*_2_,…, *p*_*k*_. Due to *u*_1_≜*u*_2_, it follows that node *u*_2_ has *k* children *q*_1_, *q*_2_,…, *q*_*k*_, and *p*_*i*_≜*q*_*i*_ (1 ≤ *i* ≤ *k*). Due to *v*_1_ ∈ *h*(*u*_1_) and *v*_2_ ∈ *h*(*u*_2_), it follows that *v*_1_ has *k* children *w*_1_, *w*_2_,…, *w*_*k*_, and *w*_*i*_ ∈ *h*(*p*_*i*_) (1 ≤ *i* ≤ *k*), *v*_2_ has *k* children *y*_1_, *y*_2_,…, *y*_*k*_, and *y*_*i*_ ∈ *h*(*q*_*i*_) (1 ≤ *i* ≤ *k*). The height of *p*_*i*_, *q*_*i*_, *w*_*i*_, and *y*_*i*_ is at most *n*. By the induction hypothesis, *w*_*i*_≜*y*_*i*_. Thus, *v*_1_≜*v*_2_.

For the “if” direction, let *v*_1_ ∈ *h*(*u*_1_), *v*_2_ ∈ *h*(*u*_2_), and *v*_1_≜*v*_2_. Similarly, we also use induction on the same height *a* of *u*_1_, *u*_2_, *v*_1_, and *v*_2_ to prove *u*_1_≜*u*_2_. If *a* = 0, that is, *v*_1_, *v*_2_ ∈ *V*_*N*_2__, and *f*_2_(*v*_1_) = *f*_2_(*v*_2_), then *u*_1_, *u*_2_ ∈ *V*_*N*_1__ and *f*_1_(*u*_1_) = *f*_2_(*v*_1_) = *f*_2_(*v*_2_) = *f*_1_(*u*_2_). Thus, in this case, *u*_1_≜*u*_2_. We assume that the result is tenable when *a* ≤ *n*, and let *a* = *n* + 1. We assume that node *v*_1_ has *k* children *w*_1_, *w*_2_,…, *w*_*k*_. Since *v*_1_≜*v*_2_, node *v*_2_ has *k* children *y*_1_, *y*_2_,…, *y*_*k*_, and *w*_*i*_≜*y*_*i*_ (1 ≤ *i* ≤ *k*). Since *v*_1_ ∈ *h*(*u*_1_) and *v*_2_ ∈ *h*(*u*_2_), *u*_1_ has *k* children *p*_1_, *p*_2_,…, *p*_*k*_, and *w*_*i*_ ∈ *h*(*p*_*i*_) (1 ≤ *i* ≤ *k*), *u*_2_ has *k* children *q*_1_, *q*_2_,…, *q*_*k*_, and *y*_*i*_ ∈ *h*(*q*_*i*_) (1 ≤ *i* ≤ *k*). The height of *p*_*i*_,  *q*_*i*_,  *w*_*i*_, and *y*_*i*_ is at most *n* by the property of node height. By the induction hypothesis, *p*_*i*_≜*q*_*i*_. Thus, *u*_1_≜*u*_2_.

Theorem 12 tells us that the semiequivalence mapping keeps the semiequivalence of nodes. Thus, all nodes in *h*(*u*) are semiequivalent. Sometimes we use *h*(*u*) to denote an arbitrary node in the set. We say that the nodes in *h*(*u*) are semiequivalent with *u*.

**Definition 13 .**

Let *N*_1_ = ((*V*_1_, *E*_1_), *f*_1_) and *N*_2_ = ((*V*_2_, *E*_2_), *f*_2_) be two networks on *𝒳*. We define the equivalence mapping between *N*_1_ and *N*_2_, *g* : *V*_1_ → *P*(*V*_2_), such that *v* ∈ *g*(*u*), for *u* ∈ *V*_1_ and *v* ∈ *V*_2_, if *v* ∈ *h*(*u*), and

(i) *u*, *v* are the roots or
(ii) node *u* has *l*  (≥1) parents *u*_1_, *u*_2_,…, *u*_*l*_; node *v* has *l* parents *v*_1_, *v*_2_,…, *v*_*l*_, and *v*_*i*_ ∈ *g*(*u*_*i*_), for 1 ≤ *i* ≤ *l*,

where *h* is a semiequivalence mapping between *N*_1_ and *N*_2_.

**Example 14 .**

Consider the networks in Figure 1. *h* is the semiequivalence mapping between *N*_1_ and *N*_2_ discussed in Example 11. *g* is an equivalence mapping between *N*_1_ and *N*_2_ defined in Definition 13. For any node *u* ∈ *V*_1_∖{*H*1, *H*2, *G*  and  *x*}, *g*(*u*) = *h*(*u*), while *g*(*v*) = *∅* when *v* ∈ {*H*1, *H*2, *G*  and  *x*}.

**Theorem 15 .**

Let *N*_1_ = ((*V*_1_, *E*_1_), *f*_1_) and *N*_2_ = ((*V*_2_, *E*_2_), *f*_2_) be two networks on *𝒳*, and let *u*_1_, *u*_2_ be two nodes in *V*_1_. *g* is an equivalence mapping between *N*_1_ and *N*_2_. Assume that *g*(*u*_1_) ≠ *∅* and *g*(*u*_2_) ≠ *∅*. Then, *u*_1_ ≡ *u*_2_ if and only if *v*_1_ ≡ *v*_2_, for *v*_1_ ∈ *g*(*u*_1_) and *v*_2_ ∈ *g*(*u*_2_).

**Proof**

Let *v*_1_ ∈ *g*(*u*_1_), *v*_2_ ∈ *g*(*u*_2_). Then *v*_1_ ∈ *h*(*u*_1_), *v*_2_ ∈ *h*(*u*_2_) based on Definition 13. For the “only if” direction, let *u*_1_ ≡ *u*_2_. We can deduce that *v*_1_≜*v*_2_ according to Theorem 12, and *u*_1_, *u*_2_ and *v*_1_ and *v*_2_ have the same depth *b*. Then, we use induction on *b* to prove that *v*_1_ ≡ *v*_2_. If *b* = 0, that is, *u*_1_, *u*_2_ are the unique root node of *N*_1_, then *v*_1_, *v*_2_ are the unique root node of *N*_2_. Thus, in this case, *v*_1_ ≡ *v*_2_. We assume that the result is tenable when *b* ≤ *n*, and let *b* = *n* + 1. We assume that node *u*_1_ has *l* parents *p*_1_, *p*_2_,…, *p*_*l*_. Due to *u*_1_ ≡ *u*_2_, node *u*_2_ has *l* parents *q*_1_, *q*_2_,…, *q*_*l*_, and *p*_*i*_ ≡ *q*_*i*_ (1 ≤ *i* ≤ *l*). Due to *v*_1_ ∈ *g*(*u*_1_) and *v*_2_ ∈ *g*(*u*_2_), *v*_1_ has *l* parents *w*_1_, *w*_2_,…, *w*_*l*_, and *w*_*i*_ ∈ *g*(*p*_*i*_) (1 ≤ *i* ≤ *l*), *v*_2_ has *l* parents *y*_1_, *y*_2_,…, *y*_*l*_, and *y*_*i*_ ∈ *g*(*q*_*i*_) (1 ≤ *i* ≤ *l*). The depth of *p*_*i*_, *q*_*i*_, *w*_*i*_, and *y*_*i*_ is at most *n* by the property of node depth. By the induction hypothesis, *w*_*i*_ ≡ *y*_*i*_. Thus, *v*_1_ ≡ *v*_2_.

For the “if” direction, let *v*_1_ ∈ *g*(*u*_1_), *v*_2_ ∈ *g*(*u*_2_), and *v*_1_ ≡ *v*_2_. We can deduce first that *u*_1_≜*u*_2_ according to Theorem 12. Similarly, we also use induction on the same depth *b* of *u*_1_, *u*_2_ and *v*_1_, *v*_2_ to prove that *u*_1_ ≡ *u*_2_. If *b* = 0, that is, *v*_1_, *v*_2_ are the unique root node of *N*_2_, then *u*_1_, *u*_2_ are the unique root node of *N*_1_. Thus, in this case, *u*_1_ ≡ *u*_2_. We assume that the result is tenable when *b* ≤ *n*, and let *b* = *n* + 1. We assume that node *v*_1_ has *l* parents *w*_1_, *w*_2_,…, *w*_*l*_. Due to *v*_1_ ≡ *v*_2_, node *v*_2_ has *l* parents *y*_1_, *y*_2_,…, *y*_*l*_, and *w*_*i*_ ≡ *y*_*i*_ (1 ≤ *i* ≤ *l*). Due to *v*_1_ ∈ *g*(*u*_1_) and *v*_2_ ∈ *g*(*u*_2_), *u*_1_ has *l* parents *p*_1_, *p*_2_,…, *p*_*l*_, and *w*_*i*_ ∈ *g*(*p*_*i*_) (1 ≤ *i* ≤ *l*), *u*_2_ has *l* parents *q*_1_, *q*_2_,…, *q*_*l*_, and *y*_*i*_ ∈ *g*(*q*_*i*_) (1 ≤ *i* ≤ *l*). The depth of *p*_*i*_, *q*_*i*_, *w*_*i*_, and *y*_*i*_ is at most *n*. So, by the induction hypothesis, *p*_*i*_ ≡ *q*_*i*_. Thus, *u*_1_ ≡ *u*_2_.

Theorem 15 tells us that the equivalence mapping keeps the equivalence of nodes. Thus, all nodes in *g*(*u*) are equivalent. Sometimes we use *g*(*u*) to denote an arbitrary node in the set. We say that the nodes in *g*(*u*) are equivalent to *u*.

**Lemma 16 .**

Let *N* = ((*V*, *E*), *f*) be a network and *u*, *v* ∈ *V* two equivalent nodes. Then *u*, *v* belong to a superconvergent set.

**Proof**

This lemma is obtained easily from Properties 1 and 2.

**Lemma 17 .**

Let *N* = ((*V*, *E*), *f*) be a partly reduced phylogenetic network. Then *u*_1_≢*u*_2_ for any two nodes *u*_1_, *u*_2_ ∈ *V*.

**Proof**

From the partial reduction procedures of the network, we have that all superconvergent sets in a partly reduced network have been deleted.

Given two networks *N*_1_ = (*V*_1_, *E*_1_) and *N*_2_ = (*V*_2_, *E*_2_), assume that *V*_1_ = {*v*_1_, *v*_2_,…, *v*_*p*_}. The unique nodes of *N*_1_, denoted by *L*(*N*_1_), is defined by the following processes. First let *L*(*N*_1_) = *∅*. Then for each one node *u* ∈ *V*_1_, if there exists no node *u*′ ∈ *L*(*N*_1_) such that *u*′ ≡ *u*, add *u* to *L*(*N*_1_). We define *L*(*N*_2_) in a similar way. Further for each node *v*_*i*_ ∈ *L*(*N*_1_), we define *e*_*N*_1__(*v*_*i*_) = |{*v* ∈ *V*_1_ : *v* ≡ *v*_*i*_}| and *e*_*N*_2__(*u*_*i*_) similarly for each node *u*_*i*_ ∈ *V*_2_. We define *e*(*∅*) = 0 for any network *N*. When the context is clear, we drop the subscript of *e*. We are now in a position to define the measure on pairs of partly reduced phylogenetic networks.

**Definition 18 .**

Let *N*_1_ = (*V*_1_, *E*_1_) and *N*_2_ = (*V*_2_, *E*_2_) be two phylogenetic networks on *𝒳*. Then *d*_*e*_(*N*_1_, *N*_2_) equals

(1) 12∑v∈LN1max⁡0,ev−ev′+∑u∈LN2max⁡0,eu−eu′,

where *v*′(*u*′) is a node in *L*(*N*_2_)(*L*(*N*_1_)) that is equivalent to *v*(*u*), and if no such equivalent node exists, then *v*′(*u*′) = *∅*.

**Lemma 19 .**

If *d*_*e*_(*N*_1_, *N*_2_) = 0 for two networks *N*_1_ = (*V*_1_, *E*_1_) and *N*_2_ = (*V*_2_, *E*_2_), then |*V*_1_ | = |*V*_2_|.

**Proof**

Let *g*_1_ : *V*_1_ → *P*(*V*_2_) and *g*_2_ : *V*_2_ → *P*(*V*_1_) be two equivalence mappings from Definition 13. Since *d*_*e*_(*N*_1_, *N*_2_) = 0, it follows that *e*(*v*_1_) = *e*(*g*_1_(*v*_1_)) (where *g*_1_(*v*_1_) denotes a node *u*, which is equivalent to *g*_1_(*v*_1_) and in *L*(*N*_2_)) along with |*g*_1_(*v*_1_)| > 0 for all *v*_1_ ∈ *L*(*N*_1_) and *e*(*v*_2_) = *e*(*g*_2_(*v*_2_)) (where *g*_2_(*v*_2_) denotes a node *u*, which is equivalent to *g*_2_(*v*_2_) and in *L*(*N*_1_)) along with |*g*_2_(*v*_2_)| > 0 for all *v*_2_ ∈ *L*(*N*_2_). From this and Theorem 15, we have that |*V*_1_ | = ∑_*v*_1_∈*L*(*N*_1_)_ *e*(*v*_1_) = ∑_*v*_1_∈*L*(*N*_1_)_ *e*(*g*_1_(*v*_1_))≤|*V*_2_| (due to *g*_1_(*v*_1_) ∈ *V*_2_) and |*V*_2_ | = ∑_*v*_2_∈*L*(*N*_2_)_ *e*(*v*_2_) = ∑_*v*_2_∈*L*(*N*_2_)_ *e*(*g*_2_(*v*_2_))≤|*V*_1_| (due to *g*_2_(*v*_2_) ∈ *V*_1_). Thus |*V*_1_ | = |*V*_2_|.

**Theorem 20 .**

Let *N*_1_ = (*V*_1_, *E*_1_) and *N*_2_ = (*V*_2_, *E*_2_) be two partly reduced networks. Then, *N*_1_ and *N*_2_ are isomorphic if and only if *d*_*e*_(*N*_1_, *N*_2_) = 0.

**Proof**

Let *g* : *V*_1_ → *P*(*V*_2_) be an equivalence mapping, as given in Definition 13. From Lemma 19, it follows that |*V*_1_ | = |*V*_2_| and *e*(*v*) = *e*(*g*(*v*)) for all *v* ∈ *L*(*N*_1_). From Lemmas 16 and 17, we have that *g*(*v*_1_) is defined and unique for each *v*_1_ ∈ *V*_1_. We now prove that if (*u*, *v*) ∈ *E*_1_, then (*u*_0_, *v*_0_) ∈ *E*_2_, where *v*_0_ = *g*(*v*) and *u*_0_ = *g*(*u*). Given that *v*_0_ = *g*(*v*), that is, *v* and *v*_0_ are equivalent, this implies that *v*_0_ and *v* have equivalent parents. Since *u*_0_ = *g*(*u*) is defined and unique, *u*_0_ is a parent of *v*_0_. Thus, (*u*_0_, *v*_0_) ∈ *E*_2_. It shows that the mapping g is bijective, which also preserves the labels of the leaves and the edges of networks. Thus, *N*_1_ and *N*_2_ are isomorphic.

The converse implication is obvious.

From the definition of the measure, the symmetry property follows immediately.

**Lemma 21 .**

For any pair networks *N*_1_ and *N*_2_, one has *d*_*e*_(*N*_1_, *N*_2_) = *d*_*e*_(*N*_2_, *N*_1_).

The measure *d*_*e*_(*N*_1_, *N*_2_) can be viewed as half of the symmetric difference of two multisets on the same set of elements, where the multiplicity of element *u* in *N*_1_ is *e*_*N*_1__(*u*) and similarly for *N*_2_. Since the symmetric difference defines a metric on multisets [12], we have the following triangle inequality.

**Lemma 22 .**

Let *N*_1_, *N*_2_, and *N*_3_ be three networks. Then, *d*_*e*_(*N*_1_, *N*_2_) + *d*_*e*_(*N*_2_, *N*_3_) ≥ *d*_*e*_(*N*_1_, *N*_3_).

From Theorem 20 and Lemmas 21 and 22, we have the following main result.

**Theorem 23 .**

The measure *d*_*e*_ is a metric on the space of partly reduced phylogenetic networks.

**Proof**

It follows from Theorem 20 and Lemmas 21 and 22 and the fact that max{0, *e*(*v*) − *e*(*v*′)} ≥ 0.

Let *N*_1_ = (*V*_1_, *E*_1_) and *N*_2_ = (*V*_2_, *E*_2_) be two phylogenetic networks. For a node *u* in *N*_1_, we refer to its semiequivalent nodes from *N*_1_ as internal semiequivalence (equivalence) nodes and its semiequivalent (equivalence) nodes from *N*_2_ as external semiequivalence (equivalence) nodes. When computing the distance between two networks, we first compute internal and external equivalence nodes for every node in the two networks; subsequently by formula (1) we obtain the distance between the two considered networks. The maximum of measure *d*_*e*_(*N*_1_, *N*_2_) is (|*V*_1_ | +|*V*_2_|)/2.0, when any node in *N*_1_ and in *N*_2_ has no external equivalence nodes.

In order to show the results of the distance computed by formula (1), we give an example as follows.

**Example 24 .**

Consider the networks in Figure 1. *N*_1_, *N*_2_ are two different networks on {1, 2, 3, 4, *x*}. However, in [11], they are indistinguishable and their *m*-distance [11] is 0. Now, we compute the *d*_*e*_-distance between them: *d*_*e*_(*N*_1_, *N*_2_) = 4 (see Example 14).

## 4. Computational Aspects

From the definition of semiequivalent nodes, whether in the same network or in two different networks, we have that the semiequivalent nodes can be computed by means of a bottom-up technique. Similarly, the equivalent nodes can be computed by means of a top-down technique. Let *N*_1_ = ((*V*_1_, *E*_1_), *f*_1_) and *N*_2_ = ((*V*_2_, *E*_2_), *f*_2_) be two phylogenetic networks. For a pair of nodes *u* and *v*, whether in the same network or in different networks, the following shows the pseudocode (Algorithm 1) that decides whether they are internal semiequivalent to each other, the pseudocode (Algorithm 2) that decides whether they are internal equivalent to each other, and the pseudocode (Algorithm 3) that computes the *d*_*e*_-distance for a pair of networks (where ISE is the abbreviation for the set of internal semiequivalent nodes, ESE is the abbreviation for the set of external semiequivalent nodes, IE is the abbreviation for the set of internal equivalent nodes, and EE is the abbreviation for the set of external equivalent nodes). If two nodes *u* and *v* from the same network are semiequivalent, then we add *u* to the ISE of *v* and add *v* to the ISE of *u*. Obviously, this decision costs at most *O*(*n* ^3^) time, where *n* = max⁡(|*V*_1_ | , |*V*_2_|). So, it takes totally *O*(*n* ^5^) time to find out all internal and external semiequivalent nodes for every node in the two networks. In a similar way, we have that it also takes *O*(*n* ^5^) time to find out all internal and external equivalent nodes for every node in the two networks. Subsequently we spend *O*(*n*) time computing the formula (1). In conclusion, it costs totally *O*(*n* ^5^) time to compute the distance between two networks, where *n* is the maximum between their node numbers.

## 5. Conclusion

In [11], Nakhleh introduced a polynomial-time computable* m*-distance in the space of reduced phylogenetic networks. In order to enlarge the space of phylogenetic networks we can compare, we devised a polynomial-time computable *d*_*e*_-distance on the space of partly reduced phylogenetic networks, which can be viewed as half of the symmetric difference of two multisets on the same set of elements. To our knowledge, the space is the largest space that has a polynomial-time computable metric. *d*_*e*_-distance is also a metric on the space of reduced phylogenetic networks which is included in the space of partly reduced phylogenetic networks. In general, for two phylogenetic networks, their *d*_*e*_-distance is larger than their* m*-distance. From [12], we have that the *d*_*e*_-distance is also a metric on the space of tree-child phylogenetic networks, semibinary tree-sibling time consistent phylogenetic networks, and multilabeled phylogenetic trees. However, the *d*_*e*_-distance is not a metric on the space of all rooted phylogenetic networks; for example, in the two phylogenetic networks in Figure 4, their *d*_*e*_-distance is 0, but they are not isomorphic.

*d*_*e*_-distance can also apply to computing the dissimilarity for other types of networks, such as spiking neural networks [18–20], which will be a direction of further research.

## Figures and Tables

**Algorithm 1:**
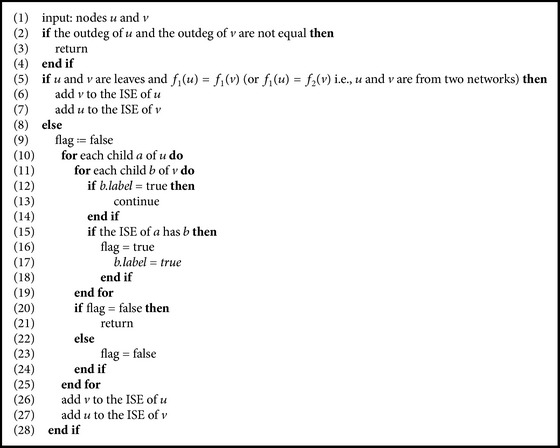
Deciding semiequivalence for two nodes *u* and *v*.

**Algorithm 2:**
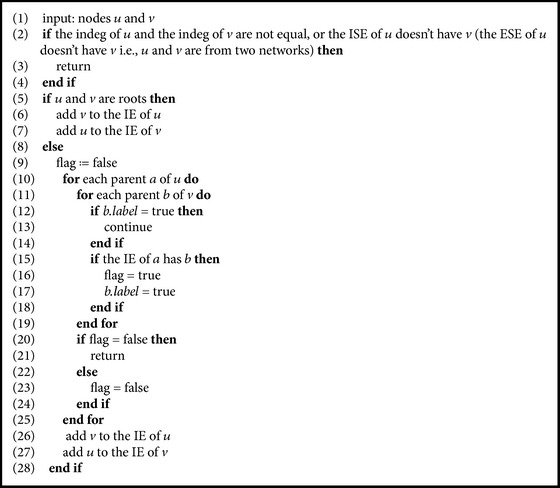
Deciding equivalence for two nodes *u* and *v*.

**Algorithm 3:**
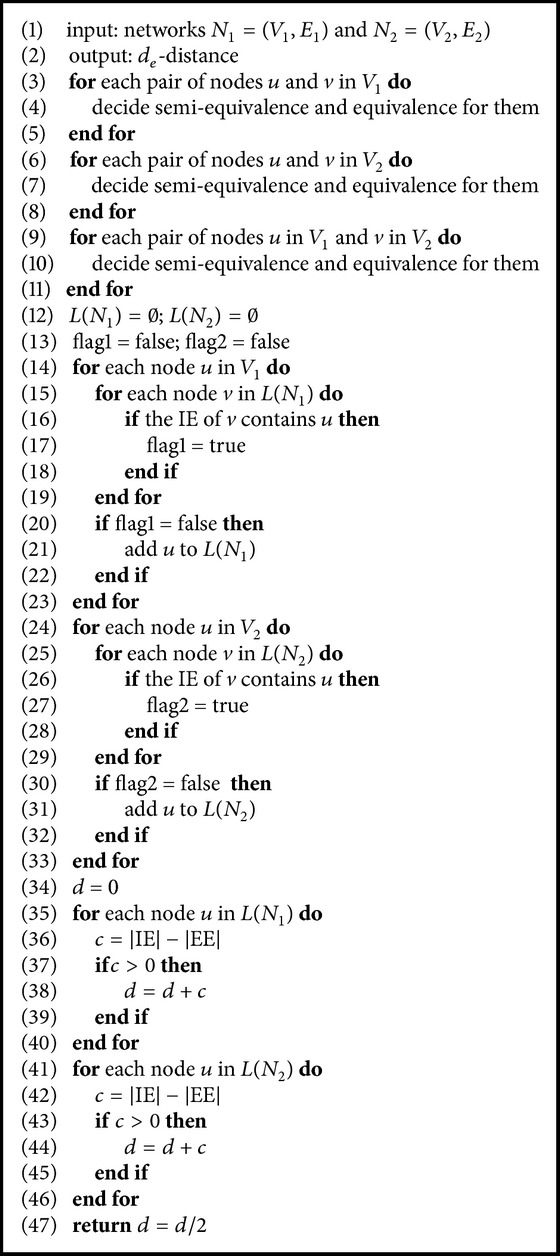
Computing the *d*_*e*_-distance for *N*_1_ = (*V*_1_, *E*_1_) and *N*_2_ = (*V*_2_, *E*_2_).
